# Soybean-derived blue photoluminescent carbon dots

**DOI:** 10.3762/bjnano.11.48

**Published:** 2020-04-09

**Authors:** Shanshan Wang, Wei Sun, Dong-sheng Yang, Fuqian Yang

**Affiliations:** 1 Department of Chemistry, University of Kentucky, Lexington, KY 40506, United States; 2 Materials Program, Department of Chemical and Materials Engineering, University of Kentucky, Lexington, KY 40506, United States; 3 College of Chemistry, Chemical Engineering and Environmental Engineering, Liaoning Shihua University, Fushun, Liaoning, 113001, China

**Keywords:** biomass, carbon dots, hydrothermal process, laser ablation, N-doping, photoluminescence

## Abstract

Biomass-derived carbon dots (CDs) are biocompatible and have potential for a variety of applications, including bioimaging and biosensing. In this work, we use ground soybean residuals to synthesize carbon nanoparticles by hydrothermal carbonization (HTC), annealing at high temperature, and laser ablation (LA) in a NH_4_OH solution. The carbon nanoparticles synthesized with the HTC process (HTC-CDs) exhibit photoluminescent characteristics with strong blue emission. The annealing of the HTC-processed carbon particles in the range of 250 to 850 °C causes a loss of the photoluminescent characteristics of the CDs without any significant change in the microstructure (amorphous structure) of the carbon particles. The LA processing of the annealed HTC-processed carbon particles introduces nitrogen-containing surface-functional groups and leads to the recovery of the photoluminescent features that are different from those of the HTC-CDs and dependent on the fraction of nitrogen in the surface-functional groups. The photoluminescence of both the HTC-CDs and LA-CDs is largely due to the presence of N-containing surface-functional groups. The quantum yield of the LA-CDs is more constant than that of the HTC-CDs under continuous UV excitation and does not exhibit a significant reduction after 150 min of excitation. The methods used in this work provide a simple and green strategy to introduce N-surface-functional groups to carbon nanoparticles made from biomass and biowaste and to produce stable photoluminescent CDs with excellent water-wettability.

## Introduction

Carbon-based quantum dots, which are referred to as carbon dots (CDs) [1] and are less-toxic and ecologically friendly [2–3], have attracted great interest due to their unique properties, such as high water solubility [4], high chemical stability [5], high photostability [6], tunable excitation and emission wavelength [7], and low cost [8]. CDs have been considered as a group of important nanomaterials with potential applications in nanotechnology [9], electrocatalysis [10], metal-ion detection [2], thermal sensing [11], drug delivery [12], and biosensing and bioimaging [1].

Several methods are available for synthesizing CDs, including oxidation and reduction [13–15], laser ablation [16], microwave irradiation [9], pyrolysis [17], and hydrothermal treatment [18]. Some of these methods are tedious and time consuming and use strong acids and/or surface treatment to improve their water solubility and luminescence properties. Hydrothermal carbonization (HTC), which can be considered as a “green technology”, has been used to produce photoluminescent CDs from biomass, including glucose, sucrose, citric acid [19], chitosan [20], orange juice [21], grass [22] and soy milk [10]. For example, Sahu et al. [21] synthesized photoluminescent CDs of 1.5–4.5 nm in diameter from orange juice at 120 °C. Liu et al. [22] produced photoluminescent polymer nanodots of 3–5 nm in diameter by using grass as a precursor at 180 °C, and Zhu et al. [10] synthesized bifunctional blue-emission carbon nanodots with diameters of 13–40 nm from soy milk also at 180 °C.

Laser ablation in liquid (LAL) has been used to produce nanomaterials with special morphologies, microstructures, and phases and with various functionalized nanostructures [23–25]. For example, carbon-based nanoparticles with fewer side-products have been synthesized from glassy carbon [16], graphite [26], polymethyl methacrylate (PMMA) [27], and a graphite–cement mixture [6] via LAL in various liquids.

In general, there are three major mechanisms contributing to the photoluminescence (PL) of CDs: 1) size-dependent bandgap (quantum confinement), 2) surface states consisting of functional groups [28], and 3) molecular state (domain size in CDs) [29–30]. Other factors, such as conjugated π-domains, molecular states, and crosslink-enhanced emission, need to be taken into account in the understanding of the PL emission of CDs. Also, the PL behavior of CDs varies with pH and temperature [18]. Wang et al. [18] studied the fluorescence of the CDs made from glucose with glutathione in a temperature range of 15 to 90 °C and observed the change of the color from dark blue to light blue and the quenching of the fluorescence at high temperatures. They attributed the fluorescent quenching to the aggregation of CDs, which increased the particle size from 2.6 ± 0.2 nm to 4.4 ± 0.2 nm. However, there are few studies in the literature focusing on the comparison of the PL behavior of CDs made from the same biomass precursor with different synthetic or processing methods; such studies would provide insight into the CD formation and PL mechanisms [31–32]. Using fluorescent N-doped CDs, which were made from grinding soybean via a pyrolysis process at 200 °C for 3 h under argon atmosphere, Xu et al. [31] observed blue emission with maximum emission of 3.17% quantum yield at ≈405 nm under 330 nm excitation. Meng et al. [32] synthesized green-emitting CDs by a hydrothermal process from soybean powder at 170 °C and obtained the highest quantum yield of 7.14% for the green-emitting CDs prepared with a processing time of 16 h.

Considering the potential applications of CDs in bioimaging and biosensing, we synthesize biocompatible CDs from soybean residuals using two strategies. The first strategy uses a one-step HTC process to produce CDs directly from the soybean residuals, and the second one uses multiple steps to produce CDs from the same soybean precursors, which involves the HTC treatment, high-temperature annealing, and LAL processing, sequentially. The purpose of the LAL processing is to introduce N-containing functional groups onto the surface of carbon nanoparticles and to recover the PL of carbon nanoparticles/CDs that was quenched by the high-temperature annealing. Both methods can be categorized as top-down methods in contrast to bottom-up methods. The PL characteristics of the CDs produced by both methods are analyzed, and the PL mechanisms of the CDs are discussed. The strategies developed in this work offer simple and effective means for producing biocompatible CDs from biomass and biowaste and manipulating their PL characteristics.

It is worth mentioning that we [33] previously studied the electrochemical performance of carbon particles of micrometer size, which were synthesized from the soybean residuals via hydrothermal carbonization and high-temperature annealing in nitrogen. In contrast to the analysis of the electrochemical performance of large carbon particles of micrometer size [33], this work is focused on the optical characteristics of nanometer-sized CDs, which are synthesized by two different approaches. One is similar to the one used for the electrochemical study and the other uses multiple processes including annealing in argon. Note that filtration is needed to obtain carbon nanoparticles from the carbonized soybean residual.

## Experimental

### Hydrothermal carbonization

Following the approach in Wang et al. [33], we hydrothermally carbonized soybean residual. Briefly, a suspension consisting of ≈40 g of ground soybean residual and ≈5 mL of 1 wt % H_2_SO_4_ aqueous solution was prepared and stirred ultrasonically. The suspension was transferred into a Teflon-lined autoclave, which was then placed in an oven for the HTC processing at 200 °C for 20 h. After the Teflon-lined autoclave was cooled to room temperature in air, filtration with filter paper of 10 μm pore size was performed to separate the HTC-produced mixture. The filtrate was further filtered with filter paper of 450 nm pore size. The material collected after the filtration was dried at 60 °C in a vacuum oven for 24 h, and the final product was named as HTC-CDs.

### High-temperature annealing

The high-temperature annealing was performed in a horizontal tube furnace under the flow of argon gas. A quartz boat loaded with HTC-processed carbon particles was placed in the tube around the center of the hot zone. The tube furnace was first flushed at room temperature with argon gas at a flow rate of 300 SCCM (standard cubic centimeters per minute) for 30 min, then heated up to a pre-set annealing temperature of 850 °C at a ramp rate of 20 °C/s under the flow of argon gas at a flow rate of 50 SCCM, and finally maintained at 850 °C for 2 h to anneal the HTC-processed carbon particles. After the annealing, the furnace/system was reduced to room temperature in air. The black carbon powders, which formed in the quartz boat, were collected, and the carbon nanoparticles obtained after filtration were named as annealed-CDs.

### Laser ablation in NH_4_OH solution

**Figure 1:**
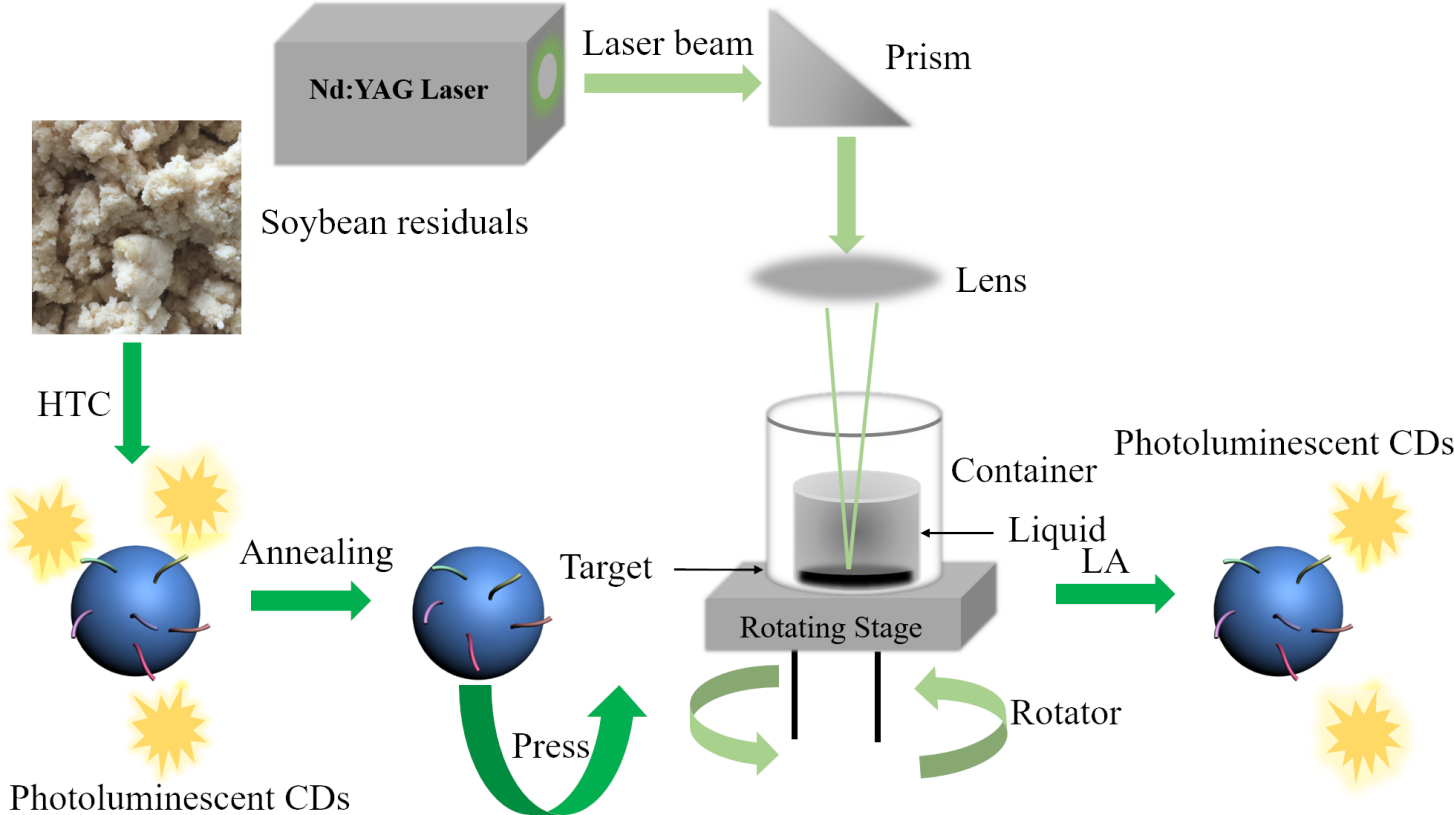
Schematic of the setup for laser ablation in liquid.

Figure 1 illustrates the process for the synthesis of CDs from ground soybean residual and the setup for the laser ablation of the annealed-HTC carbon particles in NH_4_OH aqueous solution. Using a prism and an optical lens shown in Figure 1, the beam size of the laser was adjusted to ≈2 mm. The laser wavelength was 532 nm, the pulse frequency was 50 Hz, and the dwell was 1–2 ns.

A mixture consisting of ≈0.06 g annealed-HTC carbon particles and 0.012 g Teflon powder was heated to 110 °C and maintained at 110 °C for 1 h. A mechanical press of the heated mixture at 6000 psi for 5 min led to the formation of a circular pellet of ≈1.27 cm in diameter and ≈1 mm in thickness. The circular pellet was transferred to a self-made vial with a quartz window for laser ablation, and ≈3 mL NH_4_OH aqueous solution was added to the vial to submerse the pellet to a depth of 2 cm. A quartz window lid was used to minimize the loss of the NH_4_OH solution during the laser ablation. The vial was placed on the top of the rotation stage. The rotation of the vial ensured that each laser pulse impinged on a fresh surface of the pellet.

The LAL processing of the annealed-HTC carbon particles in the NH_4_OH solution was performed with a pulse energy of 100 mJ for 1 h. After the LAL processing, the liquid that remained in the vial was collected and filtered with a 0.45 µm syringe prior to analysis. After all solvents were evaporated, ≈4 mL of DI (deionized) water was added to the vial to form a suspension, which was sonicated for 1 h at room temperature. NH_4_OH solutions of concentrations of 5%, 15%, 20% and 30% in volume were used in the LAL processing. The obtained LA samples were named as LA-CDs-x% with x representing the concentration of the NH_4_OH solution.

The control experiment of the laser-ablation of Teflon was performed following the same process as described above without any carbon materials. A circular Teflon pellet of ≈1.27 cm in diameter and ≈1 mm in thickness was prepared by the press of ≈0.06 g of pure Teflon powder at 110 °C under a compressive stress of 6000 psi for 5 min. The laser ablation of the Teflon pellet was conducted in a container with 3 mL of NH_4_OH for 1 h.

### Materials characterization

The morphology and microstructure of the prepared carbon nanoparticles were characterized on a transmission electron microscope (TEM) (JEOL 2010F). ImageJ software was used to analyze the TEM images and to determine the distribution of particle sizes for the calculation of average particle size. The X-ray photoelectron spectroscopy (XPS) analysis of the prepared carbon nanoparticles was conducted on a Thermo Scientific K-Alpha X-ray photoelectron spectrometer to determine the chemical states of elements in the prepared carbon nanoparticles.

The PL characteristics of the prepared carbon nanoparticles were characterized on a fluorometer (HORIBA, Fluoromax-3) equipped with a xenon lamp of 120 W as the light source. The absolute quantum yield (QY) was measured on a second fluorometer (Horiba Fluoromax-4) equipped with an integrating sphere. A quartz cubic cell with a 1 cm light path, which was filled with a suspension consisting of the prepared carbon nanoparticles, was used in UV–vis absorption measurements using a UV–visible spectrophotometer (Thermal Scientific Evolution 201). The Fourier-transform infrared spectroscopy (FTIR) analysis of the prepared carbon nanoparticles was conducted on a Fourier transform infrared instrument (Nicolet iS50) in the wavenumber range of 400 to 4000 cm^−1^ at a resolution of 4 cm^−1^.

## Results

**Figure 2:**
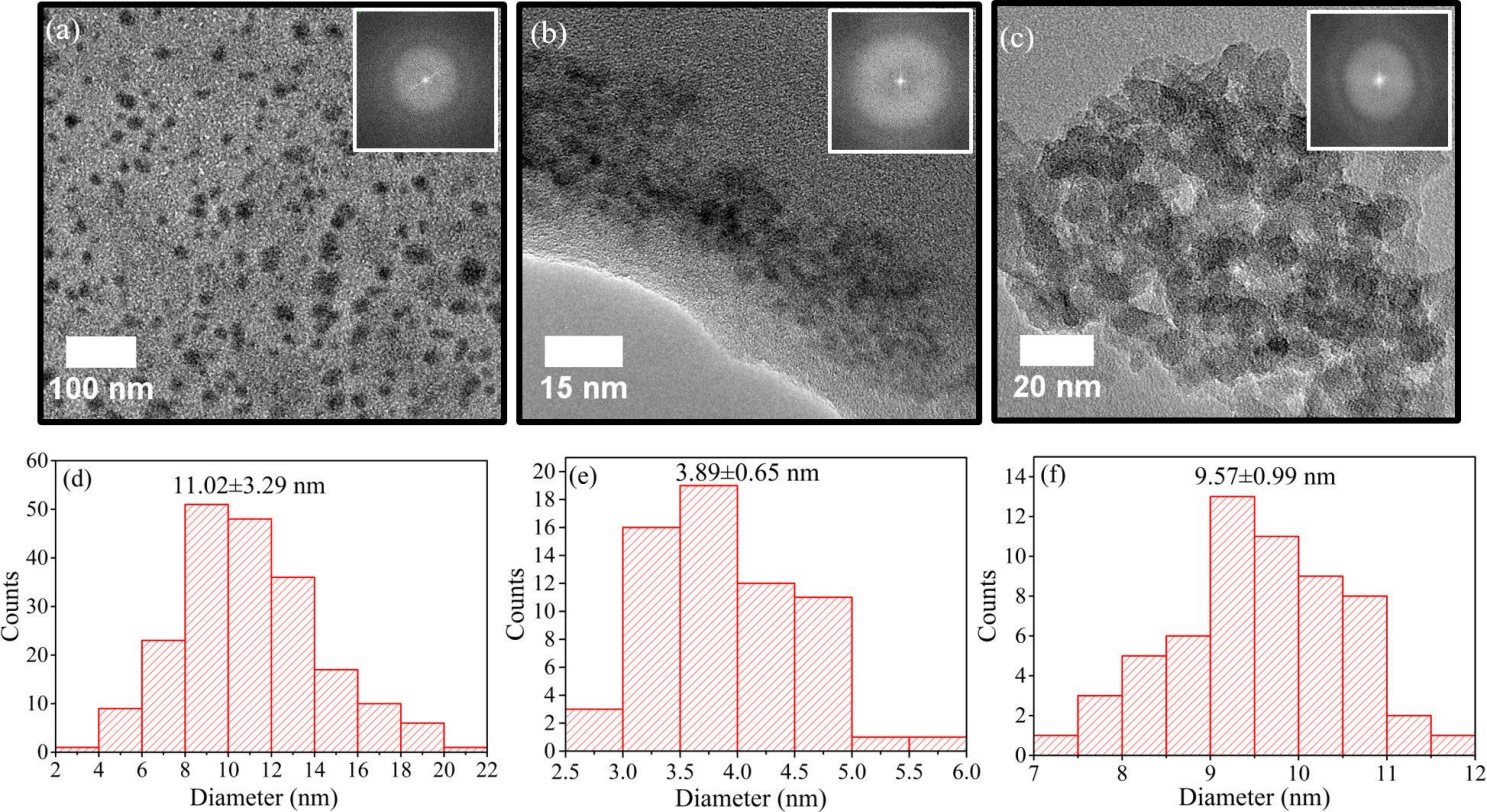
TEM images of soybean-derived nanoparticles: (a) HTC-CDs, (b) annealed-CDs (annealing temperature: 850 °C), and (c) LA-CDs-10%; the size distribution of soybean-derived nanoparticles: (d) HTC-CDs, (e) annealed-CDs, and (f) LA-CDs-10%.

Figure 2 shows TEM images of the soybean-derived nanoparticles and the corresponding size distribution. All the nanoparticles are of polygonal shape (Figure 2a–c), suggesting that the annealing at high temperatures and the laser ablation did not cause any significant changes to the morphology of the nanoparticles. The selected area electron diffraction (SAED) patterns embedded in the figures reveal that all the nanoparticles are amorphous. The EDS and XPS analyses of the HTC-CDs shown in Figure S1 and Table S1 in Supporting Information File 1 confirm that the main component of the HTC-CDs is carbon. The annealing at the temperature of 850 °C did not cause the conversion of amorphous carbon nanoparticles to nanocrystals, and the LAL processing of the annealed-HTC carbon particles also produced amorphous carbon nanoparticles.

The size distribution of the soybean-derived nanoparticles is depicted in Figure 2d–f, which was determined from the TEM images via the software of ImageJ. Slight differences are observed in the shapes of the size distribution among the HTC-CDs, annealed-CDs and LA-CDs-10%. The histogram of the HTC-CDs has a long tail in the distribution; the histogram of the annealed-CDs is close to Gaussian distribution; and the histogram of the LA-CDs-10% has a long front in the distribution. The mechanism for the differences in the shapes of the size distribution is unclear and might be due to the effects of high-temperature annealing and laser ablation on the motion of atoms. From the size distribution, we obtain the average particle sizes of the soybean-derived nanoparticles as 11.02 ± 3.29, 3.89 ± 0.65 and 9.57 ± 0.99 nm for the HTC-CDs, annealed-CDs and LA-CDs-10%, respectively. The observed average particle sizes are different from the amorphous CDs of 2.6 ± 0.2 nm made from glucose with glutathione in a temperature range of 15 to 90 °C by Wang et al. [18].

**Figure 3:**
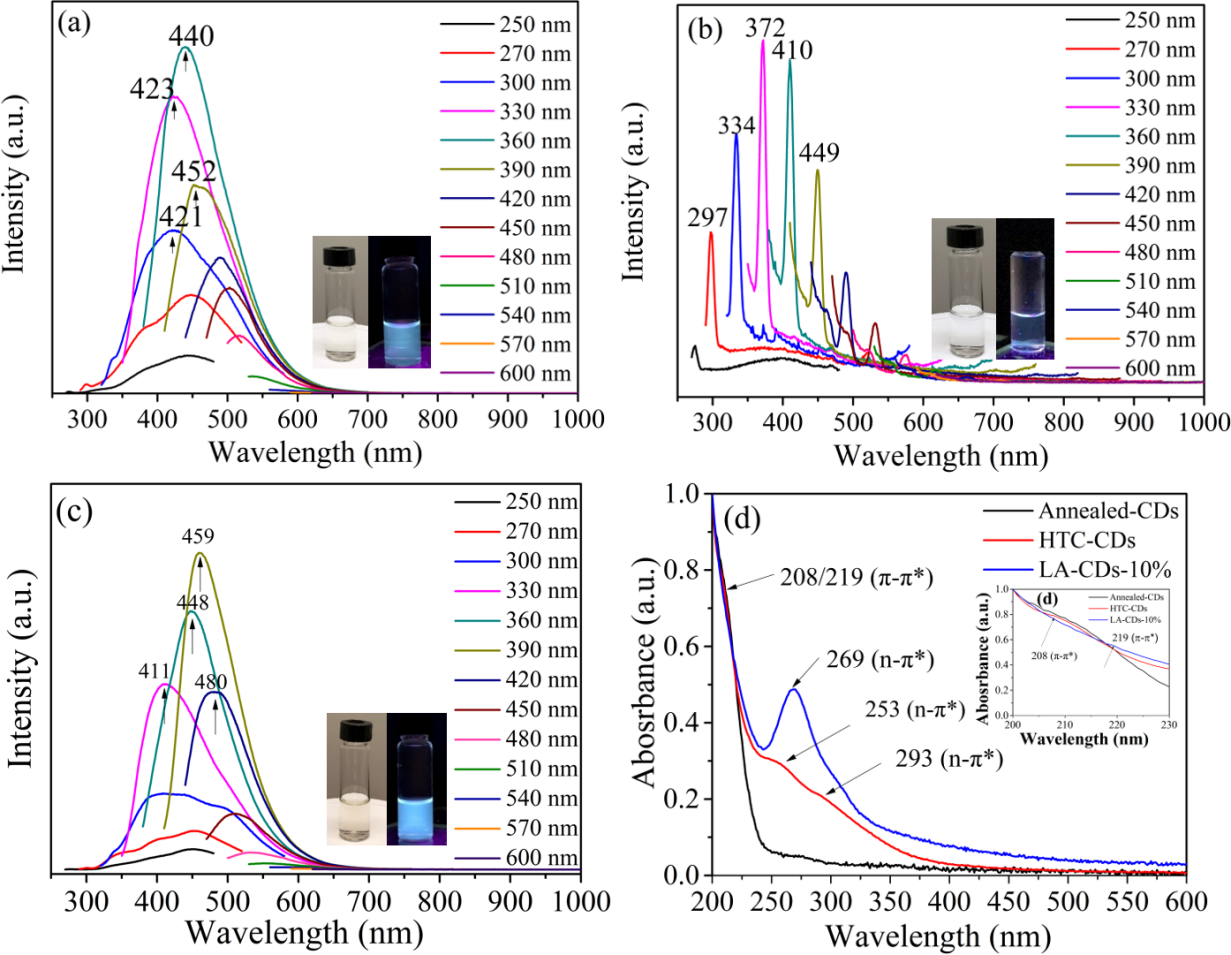
Photoluminescence spectra of: (a) HTC-CDs, (b) annealed-CDs, and (c) LA-CDs-10%; (d) UV–vis absorbance spectra of different soybean-derived CDs.

Figure 3a–c shows the PL spectra of the soybean-derived CDs. The HTC-CDs exhibited strong emission under the irradiation of UV and visible light in the wavelength range of 250 nm to 480 nm (Figure 3a), indicating the broad PL characteristics of the HTC-CDs. The broad multicolor emission can be attributed to the heterogeneity of CDs due to structural variety [1], including surface-functional groups and domain structures, and to the broad absorption ranging from UV to visible light [34]. There are three strong emission peaks at 423, 440, and 452 nm for the HTC-CDs, which are excited by the UV–vis light at the wavelengths of 330, 360 and 390 nm.

The sharp peaks presented in the PL spectrum of the annealed-CDs (Figure 3b), which are significantly different from those for the HTC-CDs and LA-CDs-10%, are due to the Raman scattering of the water [35]. The annealed-CDs show no PL under irradiation with UV–vis light. Note that the sharp peaks in Figure 3b represent the Raman scattering of water (solvent) only, indicating that there is no photoluminescence emission from the annealed-CDs under excitation at various wavelengths. The Raman scattering of the annealed CDs has a relatively low yield. The Raman peaks can be only observed when the gain or slit bandwidth of the instrument is increased to compensate for the low fluorescence signal.

In contrast to the annealed-CDs, the LA-CDs-10%, which are produced by the laser ablation of the annealed-CDs, exhibited strong emission under the irradiation of UV–vis light in the range of 250 to 480 nm. For example, the wavelength and full width at half maximum (FWHM) of the maximum emission peak are 459 nm and 91 nm, respectively, under irradiation of 390 nm light. Comparing the PL spectrum of the LA-CDs-10% with that of the HTC-CDs, we note a blue shift from 423 nm emission of the HTC-CDs to 411 nm emission of the LA-CDs-10% with 330 nm excitation.

The difference in the PL characteristics can also be observed from the insets in Figure 3a–c. The color of the aqueous solutions of both the HTC-CDs and LA-CDs-10% is light-yellow under white light and blue under UV light irradiation of 365 nm. However, there is no difference in color for the aqueous suspension of the annealed-CDs under white light and 365 nm excitation.

The UV–vis spectra of the HTC-CDs, annealed-CDs and LA-CDs-10% are depicted in Figure 3d. There are three peaks at 208, 253 and 293 nm for the HTC-CDs. The peak at 208 nm is from the π–π* transition of the conjugated C=C bond on the backbone of carbon [36], and the broad absorbance peaks at 253 and 293 nm are from the n–π* transition of the carbonyl and other oxygen or nitrogen-containing groups [37]. There is no visible peak for the annealed-CDs, which exhibited continuous, weak absorbance in the wavelength range of 200 to 600 nm. Such a result suggests that the annealed-CDs are conductive and the corresponding bandgap is zero [38].

For the LA-CDs-10%, there is a small shift of 219 nm for the peak corresponding to the π–π* transition and an increase in the intensity for the peak corresponding to the n–π* transition. The strong and broad absorption band is over the range of 240 to 350 nm with a peak at 269 nm, which can be ascribed to the complex transitions on the surface from the n–π* transitions in the groups of C=O and/or C=OOH [1,10,15]. The long tail extending to the visible spectrum can be attributed to the amino groups on the surface of the CDs [39]. Note that the LA-CDs-x% produced from the LAL processing of the annealed-CDs in the NH_4_OH solutions of different concentrations also exhibited strong absorption near 270 nm, which is attributed to n–π* transitions, as shown in Figure S4f in Supporting Information File 1. The significant differences in the wavelength and intensity of the n–π* transition between the HTC-CDs and the LA-CDs-10% suggest that the functional groups and the fractions of the functional groups on the HTC-CDs are significantly different from those on the LA-CDs-10%.

In summary, all three CDs exhibit strong π–π* absorption; the HTC-and LAL-CDs possess strong n–π* absorption, while there is no significant n–π* absorption from the annealed-CDs. Coupling the UV–vis absorption spectra with the PL spectra, we suggest that the observed PL originates from excited states produced by the n–π* excitation or from functionalized surface groups.

The time-correlated single photon counting (TCSPC) lifetime measurements were performed under 393 nm irradiation, and the emission was recorded at 440, 452, and 459 nm. The HTC-CDs exhibited the strongest emission at 440 nm, the LA-CDs-10% exhibited the strongest emission at 459 nm, and both exhibited moderate emission at 452 nm. The instrument response function was determined from the analysis of the scattering of a Ludox solution at 393 nm excitation.

**Figure 4:**
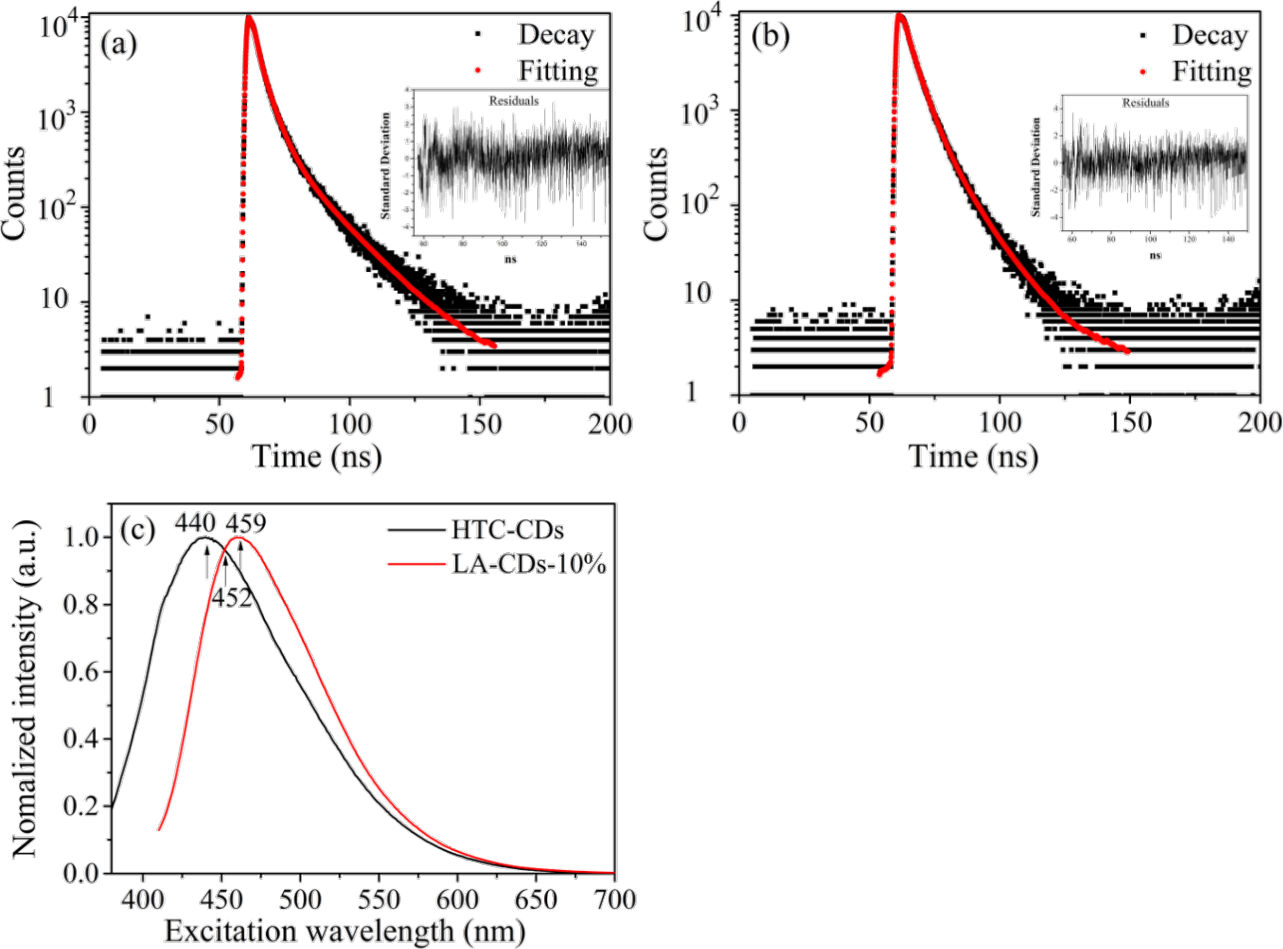
TCSPC lifetime curves under 393 nm excitation: (a) HTC-CDs at 440 nm emission, and (b) LA-CD-10% at 459 nm emission (the red lines represent the fitting curves, and the insets depict the residues for the fittings.); (c) overlaid emission spectra of the HTC-CDs and LA-CDs-10% under 393 nm excitation.

Figure 4a,b shows the PL decay curves of the HTC-CDs at 440 nm emission and the LA-CDs-10% at 459 nm emission. Assuming that the PL decay curves can be described by a triple-exponential function [38]

[1] $$N\left(t\right)=A+\sum_{i=1}^{3}B_{i}e^{-t/\tau_{i}}$$

where *N*(*t*) is the number of the photons emitted at time *t*, *A* represents the baseline/noise level, *B_i_* (*i* = 1, 2, 3) are proportionality constants for the corresponding decay functions, and τ*_i_* is the corresponding lifetime. Using Equation 1 to fit the PL decay curves in Figure 4a,b, we obtain the lifetimes of τ*_i_* and the constants of *B_i_*. The fitting curves are included in the corresponding figures. It is evident that Equation 1 is appropriate to describe the 440 and 459 nm PL-decay behavior of the HTC-CDs and the LA-CDs-10%, respectively.

Table 1 lists the lifetimes and the corresponding contributions obtained from the curve fitting. It is evident that the PL decays of both the HTC-CDs and LA-CDs-10% consist of three rate processes with the fastest one, τ_1_, attributed to the intrinsic state of the CDs, and two slower processes, τ_2_ and τ_3_, attributed to the decays of the extrinsic states associated with the surface-functional groups on the CDs [38,40]. According to Table 1, the slower process, τ_2_, contributes more than 50% to the PL decay.

**Table 1:** Lifetimes (τ_1_, τ_2_ and τ_3_) at a given emission wavelength (λ_em_) and the relative ratios (*R_i_* = *B_i_*/(*B*_1_ + *B*_2_ + *B*_3_)) (%) for the HTC-CDs and LA-CDs-10% under 393 nm excitation.

λ_ex_ = 393 nm	λ_em_ (nm)	τ_1_ (ns)	*R*_1_ (%)	τ_2_ (ns)	*R*_2_ (%)	τ_3_ (ns)	*R*_3_ (%)

HTC-CDs	440	1.21(3)	28.6	4.24(4)	52.8	15.0(1)	18.6
	452	1.05(3)	24.4	4.23(4)	55.0	13.57(8)	20.6
	459	1.14(4)	24.6	4.41(3)	55.1	14.01(7)	20.3
LA-CDs-10%	440	0.74(4)	14.0	4.13(3)	65.4	10.58(7)	20.6
	452	0.76(4)	12.3	4.18(4)	64.9	10.31(7)	22.8
	459	1.01(6)	12.2	4.38(3)	66.8	10.82(7)	21.0

**Figure 5:**
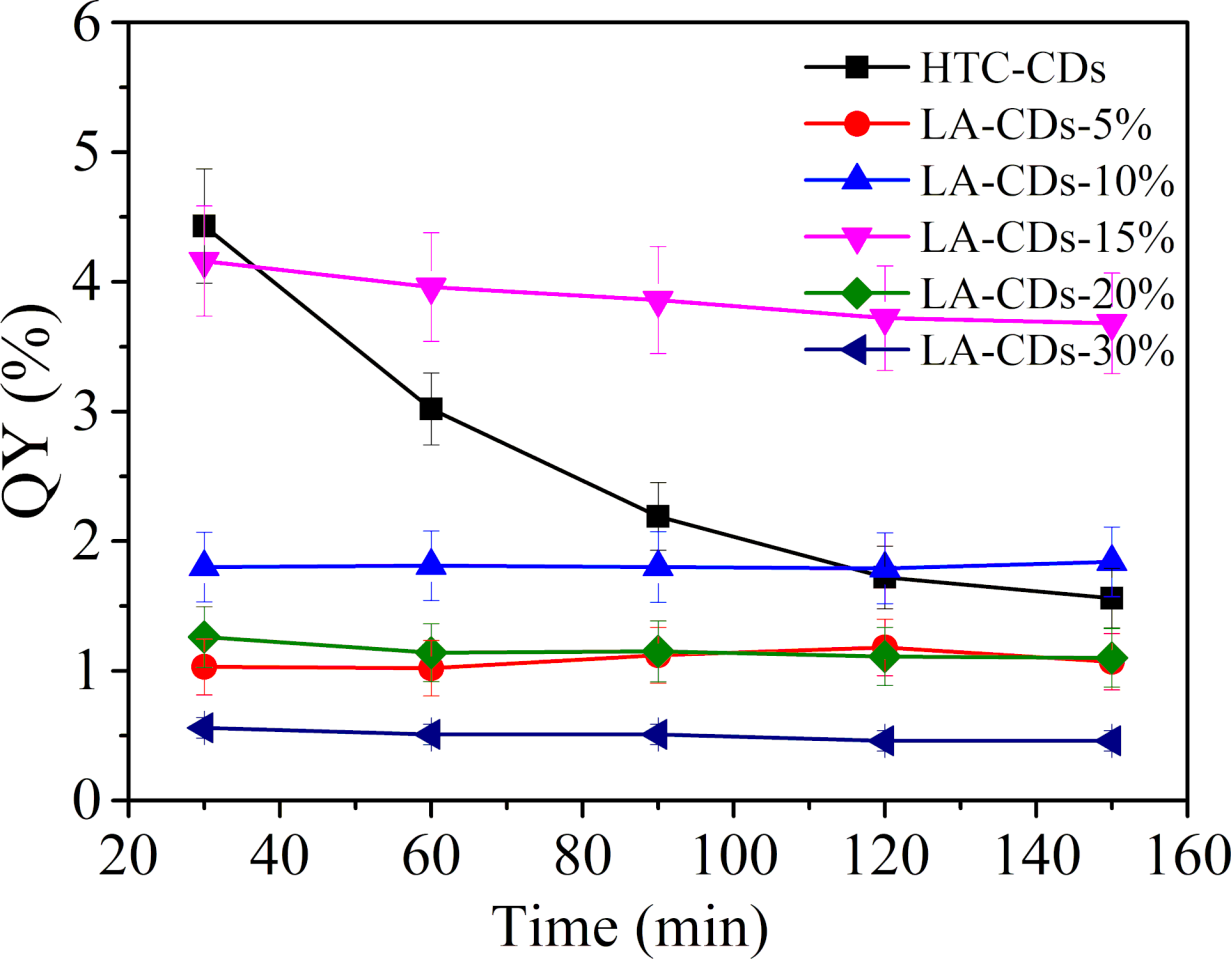
Temporal evolution of the quantum yields for the HTC-CDs under 360 nm excitation and the LA-CDs-x% under 390 nm excitation. The fluorescence intensity was calculated by integrating over the wavelength range of 340–700 nm for the HTC-CDs and 370–760 nm for the LA-CDs-x%).

Figure 5 shows the temporal variations of the QYs of the HTC-CDs under 360 nm continuous excitation and the LA-CDs-x% under 390 nm continuous excitation. Each measurement took about 30 min. The HTC-CDs displayed the largest QY initially, and the QY decreased gradually with the increase of the excitation time to 35.2% of the initial QY after continuous excitation of 150 min. Such behavior might be attributed to photobleaching associated with photoinduced changes in the structure of the functional groups on the surface of the CDs [41].

In contrast to the temporal variation of the QY of the HTC-CDs, the QY of the LA-CDs-x% is much more stable under 390 nm excitation and did not exhibit a significant reduction over the 150 min of excitation. For the LA-CDs-x% with x = 5, 10, 20, and 30, the QYs remained approximately unchanged. The LA-CDs-15% displayed the largest QY among the LA-CDs, which is compatible with the largest QY of the HTC-CDs and shows only 11.5% decrease after the 150 min of irradiation. The mecahnisms for such behavior are unclear but might be due to the relatively uniform sizes of the CDs and/or the strong bonding between the functional groups and the CDs after the LAL processing.

## Discussion

It is known that three controlling mechanisms determine the PL characteristics: 1) the domain size in CDs (molecular state), 2) the functional groups on the surface of CDs (surface state), and 3) the quantum confinement effect (size dependence). From Figure 2, we note that the average particle size of the CDs is 11.02 ± 3.29 and 9.57 ± 0.99 nm for the HTC-CDs and LA-CDs-10%, respectively, and the average particle size of the annealed-CDs is 3.89 ± 0.65 nm. There is only about 15% difference in the average particle size of the HTC-CDs and LA-CDs-10%, and the average particle size of the annealed-CDs is significantly smaller than both the HTC-CDs and LA-CDs-10%. However, the annealed-CDs did not exhibit any PL. All of these results suggest that the size effect (quantum confinement effect) is negligible. The PL characteristics of the soybean-derived CDs are dependent on the domain size in CDs (molecular state) and the functional groups on the surface of CDs (surface state).

Table 2 summarizes the PL characteristics of the HTC-CDs, annealed-CDs and LA-CDs-10% under 330, 360 and 390 nm excitation, respectively. All the annealed-CDs in the annealing temperature range of 250 to 850 °C did not exhibit any PL characteristics under excitation at any of the three wavelengths. With the annealing temperature in the range of 250 to 850 °C, there is no significant change in the microstructure (amorphous structure) of carbon nanoparticles, suggesting that annealing did not likely cause any significant changes to the domain sizes in the HTC-CDs. Note that the annealing did cause the change in the size distribution of the carbon nanoparticles, as revealed in Figure 2e. Thus, it is the functional groups on the surface of the CDs that play the key role in the light emission of the soybean-derived CDs under the excitation of UV light.

**Table 2:** PL characteristics of the synthesized CDs.^a^

Excitation wavelength (nm)	HTC-CDs		Annealed-CDs		LA-CDs-10%	
	Emission peak (nm)	FWHM (nm)	Emission peak (nm)	FWHM (nm)	Emission peak (nm)	FWHM (nm)

330	423 ± 3	129	NA		411 ± 3	122
360	440 ± 1	109	NA		448 ± 2	203
390	452 ± 3	109	NA		459 ± 2	91
QY (%)	4.46		NA		1.81	

^a^FWHM = full width at half maximum, NA = not available.

Comparing the PL characteristics of the HTC-CDs with those of the LA-CDs-10%, we note the differences in the emission wavelength and the FWHM. Such differences reveal that the functional groups on the surface of the HTC-CDs are likely different from those on the surface of the LA-CDs-10%. Annealing the HTC-CDs damaged/destroyed the surface structures/function groups, resulting in the complete loss of the PL characteristics. The LAL processing of the annealed-HTC carbon particles in the NH_4_OH aqueous solution introduced “new” functional groups to the surface of the CDs, leading to the “re-birth” of the PL characteristics. It is interesting to note that there are large fluctuations in the FWHM for the LA-CDs-10%, which is likely attributed to the fluctuations of the bandgap associated with the functional groups and the disorder of crystallinity introduced by the LAL processing.

**Figure 6:**
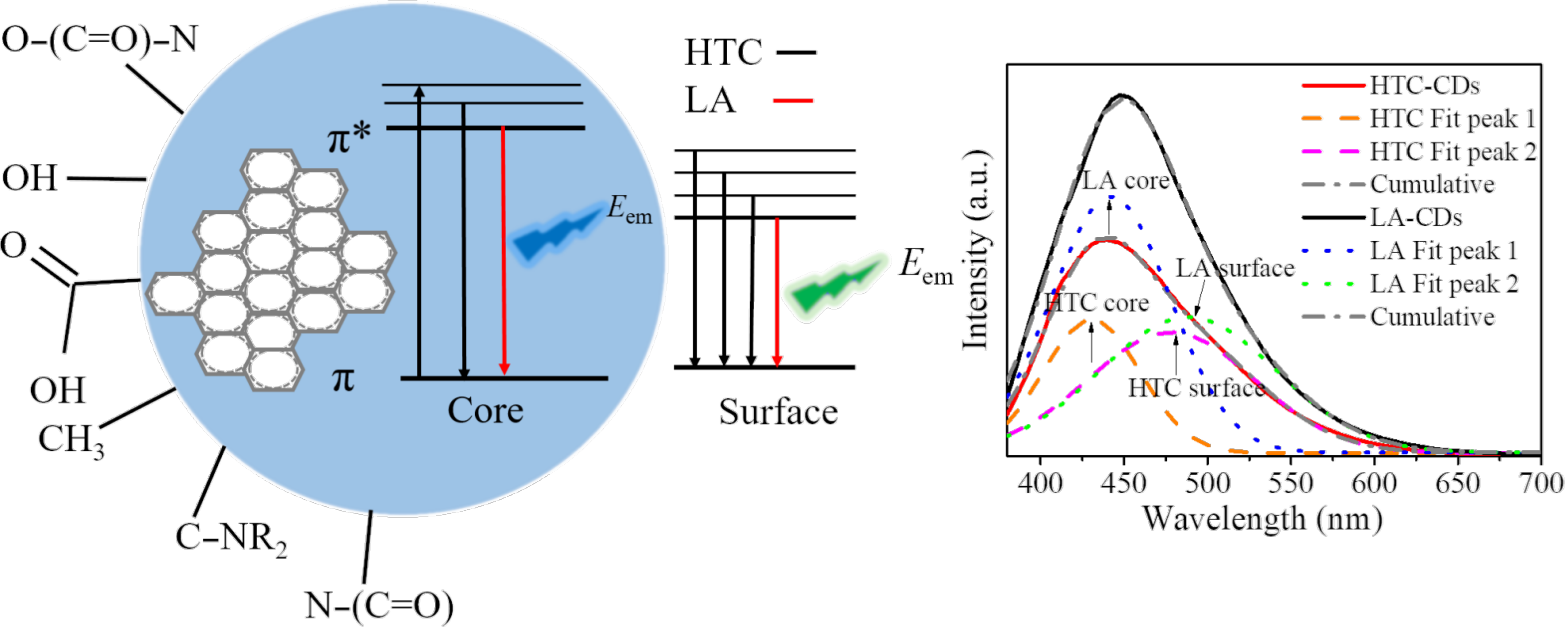
Schematic of the bandgaps of CDs under 360 nm excitation and the deconvolution bands (fitted with a two-Gassian function).

According to Yu et al. [42], the emission peak of CDs can be fitted with a two-Gaussian function, associated with the “core” state and “surface” state, as shown schematically in Figure 6. From Figure 3, we note that the strongest emssion is at 440 nm for the HTC-CDs and 459 nm for the LA-CDs-10%. From Figure 6, we note that both peaks indeed can be fitted with a two-Gassian function, respectively. The fitting results reveal that the 440 nm emission peak of the HTC-CDs can be fitted by a two-Gaussian function: one with a high energy band (the “core band”) has a peak at 430 nm and a FWHM of 69 nm, and the other with a low high energy band (the “surface” band) has a peak at 478 nm and a FWHM of 118 nm; and the 450 nm emssion peak of the LA-CDs-10% can be fitted by a two-Gaussian function: the “core” band has a peak at 442 nm and a FWHM of 83 nm, and the “surface” band has a peak at 488 nm and a FWHM of 124 nm.

By integrating both the “core” band and the “surface” band, respectively, we obtain the area ratios of the “surface” band to the “core” band as 1.6 for the HTC-CDs and 1.0 for the LA-CDs-10%. Such a difference in the area ratios (1.6 vs 1.0) reveals the important role of the “surface” states and surface-functional groups in controlling the PL characteristics of the soybean-derived CDs. The FWHM difference between the strongest emission peaks of the HTC-CDs and LA-CDs-10% is likely attributed to the difference in the “surface” states and surface-functional groups.

Two possibile mechanisms contribute to the loss of the PL characteristics of the annealed-CDs: the damage/destruction of the “surface” states/functional groups and the irreversible change in the energy gap induced by the annealing. It is known that the temperature dependence of the energy gap for the fluoresence of CDs can be expressed as [42]

[2] $$E_{\text{g}}\left(T\right)=E_{\text{g}}\left(0\right)-2S<h\omega >{\left(e^{<h\omega >/kT}-1\right)}^{-1}$$

where *E*_g_(*T*) is the energy gap at temperature *T*, *S* is the Huang–Rhys factor representing the coupling strength between exciton and phonon, <*h*ω> is the phonon energy, and *k* is the Boltzmann constant. It is evident that increasing temperature leads to the decrease of the energy gap. The CDs become conductive at temperatures larger than the critical temperture and lose the PL charactersistic, as demonstrated by the annealed-CDs due to the irreversible change in the energy gap at high temperatures.

**Figure 7:**
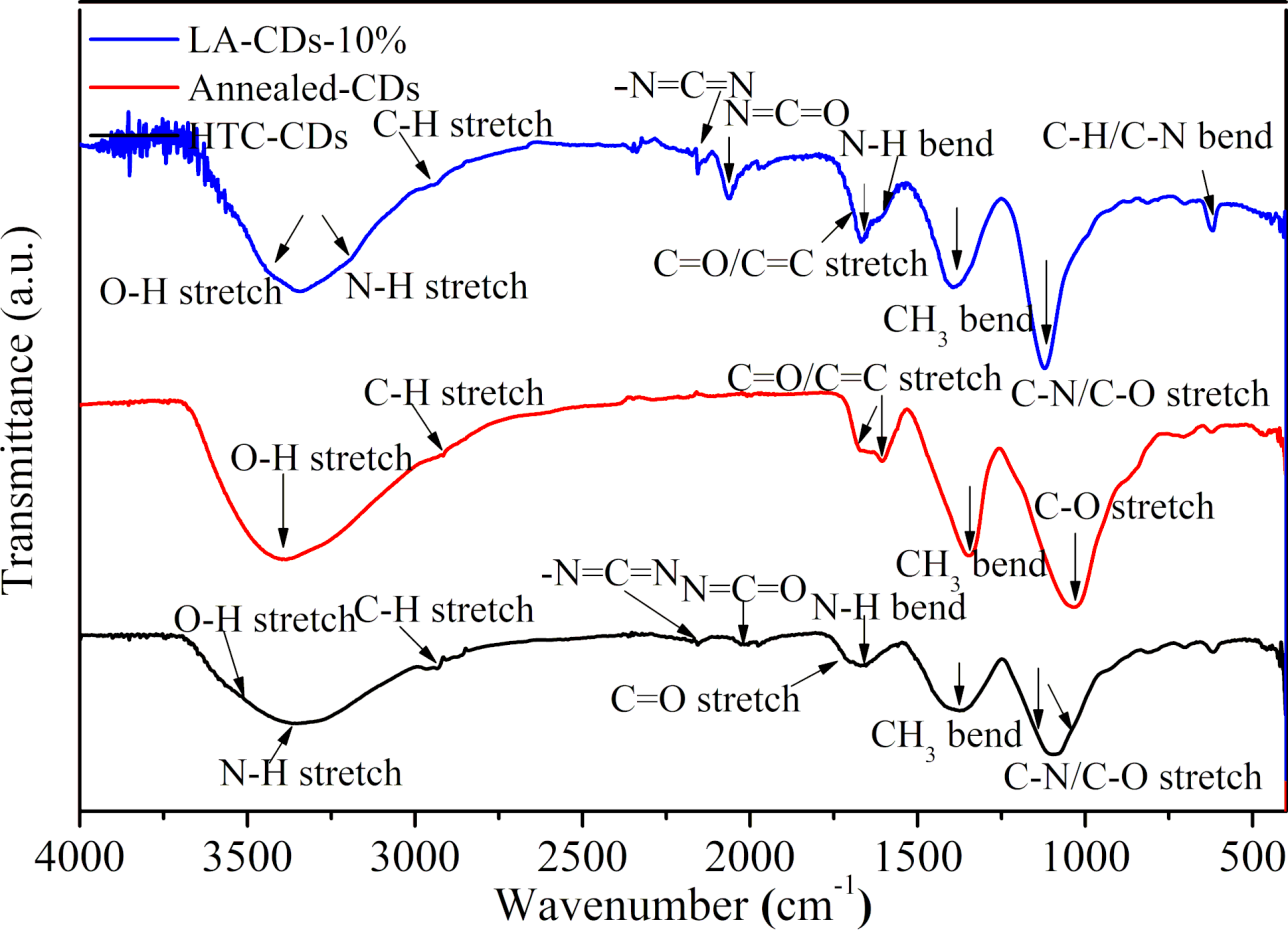
FTIR spectra of soybean-derived carbon nanoparticles.

The PL characteristics of CDs depend on the structure and composition of the CDs, as shown by the FTIR spectra of the HTC-CDs, annealed-CDs, and LA-CDs-10% (Figure 7). The FTIR spectrum of the HTC-CDs is similar to that of LA-CDs-10%, suggesting the presence of similar chemical-bonding structures. For example, both CDs possess the bonds of O–H and N–H stretching vibration at 3344 cm^−1^ and 3366 cm^−1^ [43–44], respectively. The presence of the O–H and N–H groups makes the CDs hydrophilic and improves the stability and dispersibility of the CDs in aqueous solutions. In addition, there are –N=C=N, N=C=O stretching vibrations represented by the weak bonds at 2156 and 2023 cm^−1^ for the HTC-CDs and 2155 and 2063 cm^−1^ for the LA-CDs-10% [45]. The C–H stretching vibrations are at 2931 and 2875 cm^−1^, respectively [7]. The stretching bonds of the C=O/C=C bond are around 1706 and 1670 cm^−1^, respectively [46], and the CH_3_ bending vibrations are at 1375 and 1389 cm^−1^, respectively [38]. The stretching bonds of the C–N/C–O bond are at 1096 and 1117 cm^−1^ for the HTC-CDs and the LA-CDs-10%, respectively [38,44]. Also, there are weak bonds in the range of 800–600 cm^−1^, corresponding to the bending vibrations of C–O and C–N bonds. Note that the FTIR spectra of all the LA-CDs-x% reveal the presence of N-containing groups, as shown in Figure S3f in Supporting Information File 1, suggesting that the LAL processing of the annealed-HTC carbon particles introduced N-based functional groups on the surface of the CDs.

In contrast to the HTC-CDs and the LA-CDs-x%, the FTIR spectrum of the annealed-CDs does not have peaks in the range of 2165–2115 cm^−1^ and 2115–1988 cm^−1^, which are associated with the presence of nitrogen. Such a difference reveals the importance of nitrogen in controlling the PL characteristics of the soybean-derived CDs. It is the N-containing functional groups that determine the PL behavior of both the HTC-CDs and LA-CDs-x%. Note that there is a new peak around 1600 cm^−1^ for the annealed-CDs, which is attributed to aromatic C=C ring bond [47–48].

**Figure 8:**
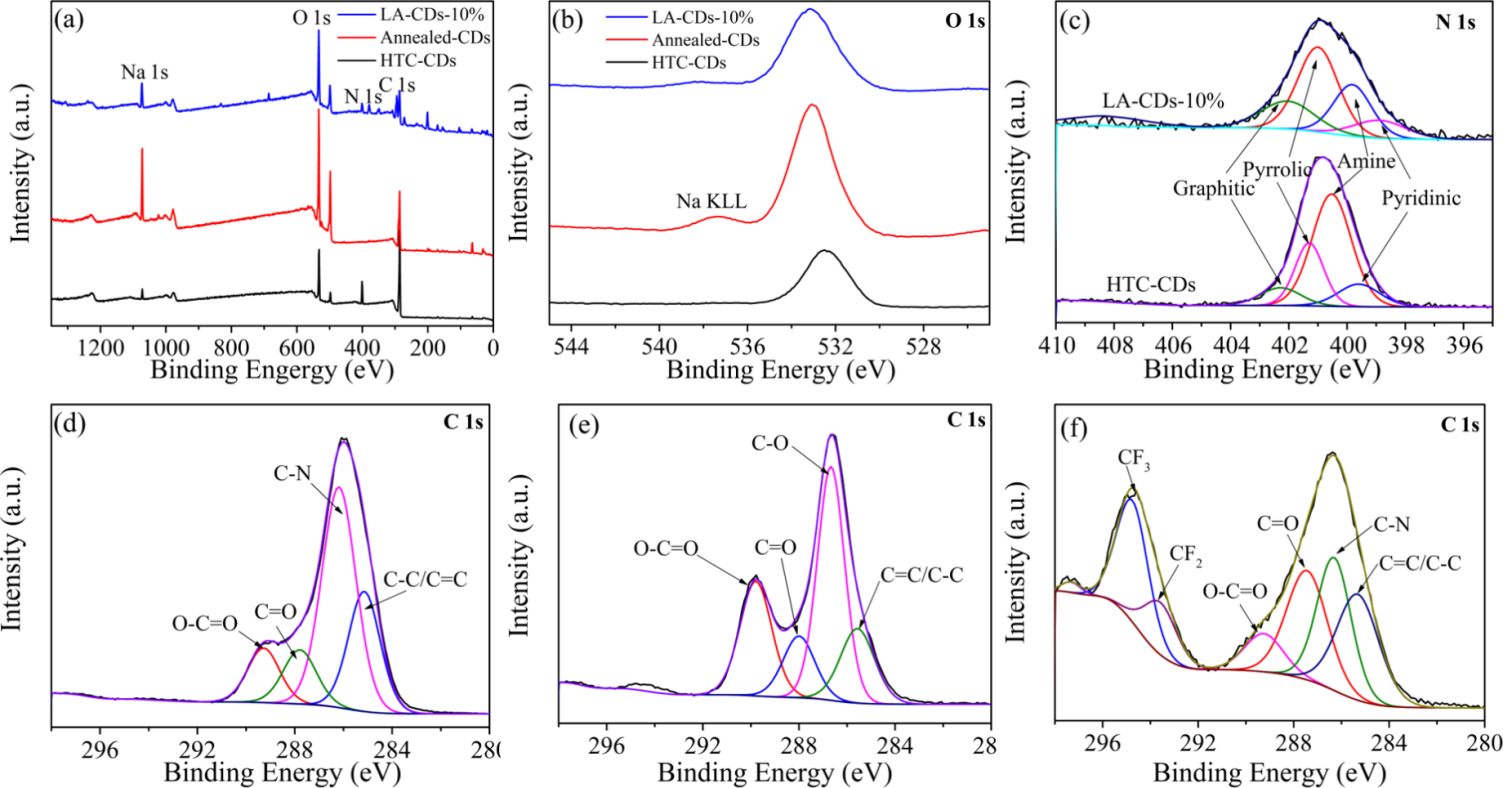
(a) XPS survey spectra, (b) deconvoluted high-resolution spectra of O 1s, (c) deconvoluted high-resolution spectra of N 1s; and deconvoluted high-resolution spectra of C 1s of (d) HTC-CDs, (e) annealed-CDs, and (f) LA-CDs-10%.

As discussed above, the PL characteristics of the soybean-derived CDs are dependent on the surface-functional groups. XPS analysis was performed to determine the chemical states of elements on the surface of the soybean-derived C-dots. Figure 8 depicts the XPS spectra of the soybean-derived CDs. Table S1 in Supporting Information File 1 lists the chemical compositions on the surface of the soybean-derived CDs, as determined from the XPS analysis. Both the HTC-CDs and LA-CDs-10% contain the elements C, N and O: 10.3% of N, 16.1% of O and 73.6% of C in the HTC-CDs and 4.4% of N, 44.5% of O and 51.1% of C in the LA-CDs-10%. The ratios of N to C and O to C are 0.14 and 0.08, respectively, for the HTC-CDs and 0.09 and 0.87, respectively, for the LA-CDs-10%. The HTC-CDs are relatively N-rich, and the LA-CDs-10% are O-rich. The differences in the fractions of oxygen and nitrogen likely suggest that the two kinds of the CDs might possess different surface-functional groups, leading to the distinct PL responses under UV–vis irradiation. According to Figure 8a and Table S1 in Supporting Information File 1, there is no nitrogen present in the annealed-CDs, in accord with the FTIR measurement shown in Figure 7. This result confirms again the important role of nitrogen in the PL response of the soybean-derived CDs.

Figure 8b–f shows the deconvoluted high-resolution spectra of O 1s, N 1s and C 1s in the soybean-derived CDs. The binding energies of O 1s are 532, 533, and 533 eV for the HTC-CDs, annealed-CDs and LA-CDs-10%, respectively, which are deconvoluted to C–O and C=O bonds. For the HTC-CDs, the binding energies of N 1s determined from the deconvoluted high-resolution spectrum of N 1s are 398.1, 399.3, 401.5, and 402.1 eV, which are assigned to pyridinic, amine, pyrrolic and graphitic nitrogen, respectively [49–50]. For the LA-CDs-10%, the binding energies of the corresponding N 1s are 398.7, 399.6, 401.0 and 402.1 eV, respectively. Thus, both of the HTC-CDs and the LA-CDs-10% likely possess the same types of surface-functional groups but with different fractions. The difference in the fractions of the surface-functional groups leads to different PL characteristics.

For the HTC-CDs, the binding energies of C 1s are measured to be 285.1, 286.2, 287.8, and 289.3 eV, which are assigned to the C–C/C=C (sp^3^ and sp^2^), C–N (sp^3^), C=O, and O–C=O (sp^2^), respectively [20,38,51–52]. The binding energies of C 1s in the annealed-CDs are 285.6, 286.7, 287.9 and 289.8 eV, corresponding to the C–C/C=C, C–O, C=O and O–C=O groups, while the binding energies of C 1s in the LA-CDs-10%, are 294.8, 293.6, 289.3, 287.4, 286.3, and 285.3 eV, corresponding to the C–F_3_, C–F_2_, O–C=C, C=O, C–N, and C–C/C=C groups, respectively [53]. The presence of the C–F_3_ bond in the LA-CDs-10% was from the Teflon binder used for the LAL processing. Note that the Teflon binder has no effect on the emission, as revealed in Figure S5 in Supporting Information File 1. According to Figure S5, the sharp peaks in the photoluminescence spectrum are from the water Raman scattering. The broad peaks of the emissions at 418, 422 and 436 nm have much weaker intensities than the water Raman scattering, suggesting that the PL intensity from laser-ablated Teflon is negligible. Thus, both the HTC-CDs and the LA-CDs-10% possessed C–N groups, which contributed to the PL response under UV excitation.

Additional peaks at 1074.3, 687.1, 533.1, 400.8 and 286.4 eV in the XPS spectrum of the LA-CDs-10% are attributed to Na 1s, F 1s, O 1s, N 1s and C 1s. The presence of the Na 1s peak was due to the sodium residual present in the glassware.

The XPS analyses of the LA-CDs-x% were also performed, as shown in Figures S2 and S3 and summarized in Tables S2 and S3 in Supporting Information File 1. Similar to the LA-CDs-10%, all the other LA-CDs-x% exhibited a strong N 1s peak in the range of 400–402 eV, corresponding to the graphitic, pyrrolic, amine and pyridinic nitrogen. All these results suggest that the LAL processing of the annealed-HTC carbon particles in NH_4_OH solutions is an effective method for doping N into carbon nanoparticles, and the amount of doped N can be simply controlled by the concentration of the NH_4_OH solution.

According to Figure 4a,b, the PL decay exists for the 440 and 450 nm emissions of the HTC-CDs and LA-CDs-10%, respectively, under 393 nm excitation. Fitting the data to a triple-exponential function, we determined the lifetimes for the corresponding rate processes and the relative contributions, as listed in Table 1.

For the HTC-CDs, the strongest emission is at the wavelength of 440 nm. The lifetime of the intrinsic state (τ_1_) at 440 nm emssion was 1.21 ns with a relative contribution of 28.6%. The lifetimes and the relative contributions of the intrinsic state (τ_1_) are (1.05 ns, 24.4%) and (1.14 ns, 24.6%) for emission at longer wavelengths of 452 and 459 nm, respectively. For the 440 nm emission, the lifetimes and the relative contributions of the surface-functional groups (τ_1_ and τ_2_) are (4.24 ns, 52.8%) and (15.0 ns, 18.6%), respectively; for the 452 and 459 nm emissions, the lifetimes and the relative contributions of the surface-functional groups are ((4.23 ns, 55.0%), (13.6 ns, 20.6%)) and ((4.41 ns, 55.1%), (14.0 ns, 20.3%)), respectively. It is evident that the surface-functional groups played a dominant role in determining the lifetime of the PL response of the HTC-CDs [54].

From Table 1, we note that the strongest emission for the LA-CDs-10% is present at 459 nm, and the lifetimes for the intrinsic state and the surface-functional groups are generally lower than those for the HTC-CDs under UV excitation of the same wavelengths. On the other hand, the relative contributions of the surface-functional groups are larger than those for the HTC-CDs. That is to say, there is more contribution from the surface-functional groups in the LA-CDs-10% than those in the HTC-CDs. The LAL processing of the annealed-CDs likely increases the fraction of the surface-functional groups responsible for the PL response of the soybean-derived CDs.

According to the above discussion, we can conclude that the N-containing functional groups play the key role in determining the PL response of the soybean-derived CDs. The LAL processing of the annealed-HTC carbon particles in the NH_4_OH solution successfully introduced the surface-functional groups with nitrogen to the CDs, and all the LA-CDs-x% exhibited PL emission under UV excitation over a wide range of wavelengths. Under 390 nm excitation, the wavelengths corresponding to the maximum emission peaks are 464, 459, 463, 462, and 454 nm respectively for the LA-CDs-5%, LA-CDs-10%, LA-CDs-15%, LA-CDs-20% and LA-CDs-30% (Figure S4, Supporting Information File 1). There is no significant difference in the wavelengths corresponding to the maximum emission peaks. However, there exists a dependence of the QY of the PL emission on the fraction/amount of nitrogen.

**Figure 9:**
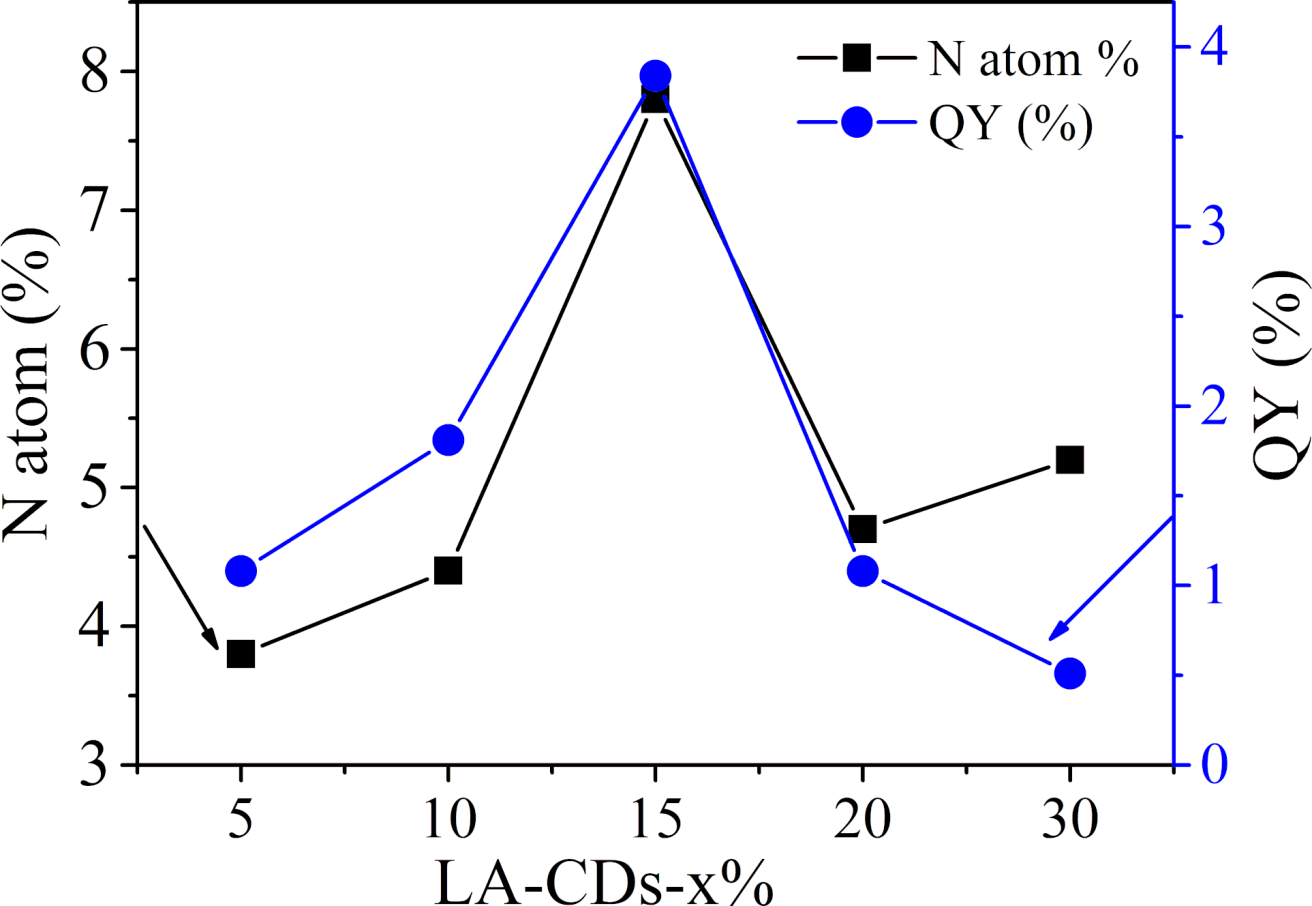
Variations of atomic fraction of N and QY of LA-CDs-x% with the concentration of NH_4_OH.

Figure 9 shows the variations in the atomic fraction of N and QY of the LA-CDs-x% with the concentration of NH_4_OH. Both the atomic fraction of N and QY of the LA-CDs-x% increase first with increasing concentration of NH_4_OH, then reach the maximum, and then decrease with increasing concentration of NH_4_OH. In general, the QY of the LA-CDs-x% is a function of the fraction/amount of nitrogen, which is consistent with the results reported in the literature [54]. There exists a maximum fraction of nitrogen, which can be introduced through the LA-activated surface-functional groups to the annealed-CDs. Note that the deviation of the QY trend from that of the N-atomic fraction at high N content implies that other factors (i.e., defects) may start to influence the PL response of the LA-CDs-x% with x ≥ 30%.

Table 3 summarizes the PL characteristics of the soybean-derived CDs. For comparison, the results obtained by Xu et al. [31] and Meng et al. [32] are also included in Table 3. In general, all the soybean-derived CDs exhibited different PL characteristics, as revealed by the difference in the wavelength of the maximum emission for different excitations. The QYs of the soybean-derived CDs obtained in this work are comparable to the result given by Xu et al. [31] and smaller than the result given by Meng et al. [32]. All of these works reveal the important role of the structure (surface state and molecular state) of the soybean-derived CDs, which is dependent on the carbonization process and the processing parameters, in controlling the optical properties of the biomass-derived CDs.

**Table 3:** PL characteristics of the soybean-derived CDs.

Processing method	Temperature (°C)	Agent	Time (h)	Maximum emission/excitation wavelength	Atomic fraction of C, O and N (%)	QY (%)

HTC-CDs (this work)	200	1 wt % H_2_SO_4_	2	440/360	73.6/16.1/10.3	4.46
annealed-CDs (this work)	850	argon	2	N/A	60.0/40.0/0.0	N/A
LA-CDs (this work)	25	15 vol % NH_4_OH	1	459/390	54.7/37.5/7.8	3.84
pyrolysis [31]	200	argon	3	405/330	81.28/15.92/2.49	3.17
HTC [32]	170	DI water	16	≈533/470	46.27/≈/≈	7.14

## Conclusion

In summary, we have synthesized carbon nanoparticles with different PL characteristics from the ground soybean residual via the combination of hydrothermal carbonization, annealing at high temperature and laser ablation in NH_4_OH solution. The annealing of the soybean-derived carbon particles at high temperature damaged/destroyed the surface structures of carbon nanoparticles, removed the nitrogen-containing surface functional groups, and resulted in the loss of the PL characteristics. The laser ablation of the annealed soybean-derived carbon particles in the NH_4_OH solutions introduced N-containing surface-functional groups on the carbon nanoparticles.

Both the HTC-CDs and the LA-CDs-x% exihibited PL characteristics under UV–vis excitation due to the presence of N-containing surface-functional groups, even though the PL response under UV excitation of the same wavelength was different. The difference in the PL response can be attribued to different amounts of N-containing surface-functional groups, even though both of the soybean-derived nanoparticles possessed similar types of surface-functional groups.

The results presented in this work reveal the important role of the surface-functional groups in controlling the PL response of CDs and shed insight into the PL mechanisms of the CDs with N-containing surface-functional groups. This work offers a simple, green, and effective strategy for the surface N-functionalization of carbon nanoparticles derived from biomass and biowaste and for the production of carbon nanoparticles with stable PL characteristics and excellent water-wettability.

## Supporting Information

File 1 Additional experimental data.
