# Supplementary material for: Soybean-derived blue photoluminescent carbon dots
Source: Beilstein J Nanotechnol. 2020 Apr 9;11:606–19. doi: 10.3762/bjnano.11.48 (PMC7155895; doi:10.3762/bjnano.11.48)
Supplement: File 1 — Additional experimental data. [file Beilstein_J_Nanotechnol-11-606-s001.pdf]

BEILSTEIN JOURNAL OF NANOTECHNOLOGY

Supporting Information

for

Soybean-derived blue photoluminescent carbon dots

Shanshan Wang, Wei Sun, Dong-sheng Yang and Fuqian Yang

Beilstein J. Nanotechnol. **2020,** *11,* 606–619. doi:10.3762/bjnano.11.48

Additional experimental data

License and Terms: This is a supporting information file under the terms of the Creative Commons Attribution License (http://creativecommons.org/licenses/by/4.0). Please note that the reuse, redistribution and reproduction in particular requires that the authors and source are credited.
The license is subject to the *Beilstein Journal of Nanotechnology* terms and conditions: (https://www.beilstein-journals.org/bjnano)

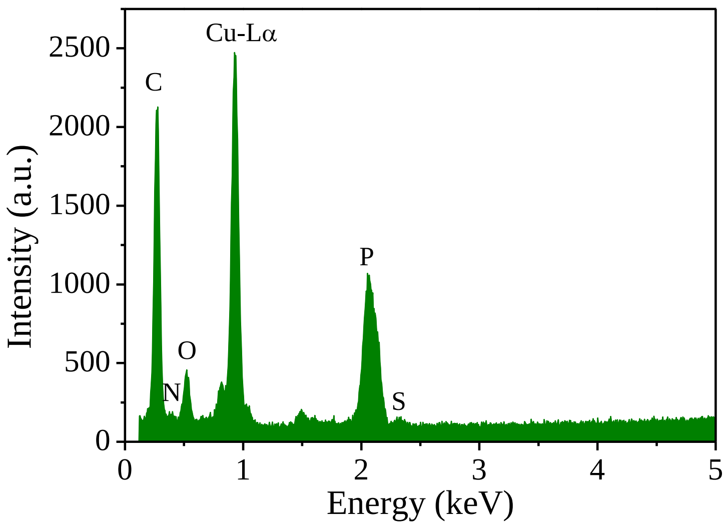

Figure S1: EDX spectrum of HTC-CDs.

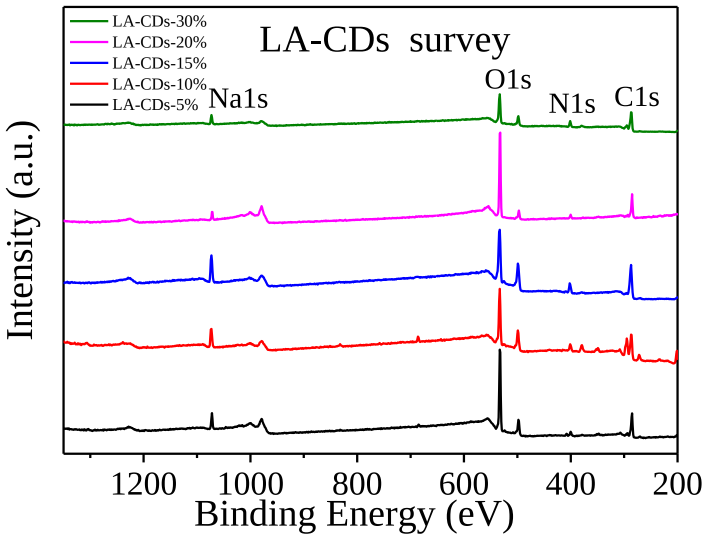

Figure S2: XPS survey spectra of LA-CDs-x%.

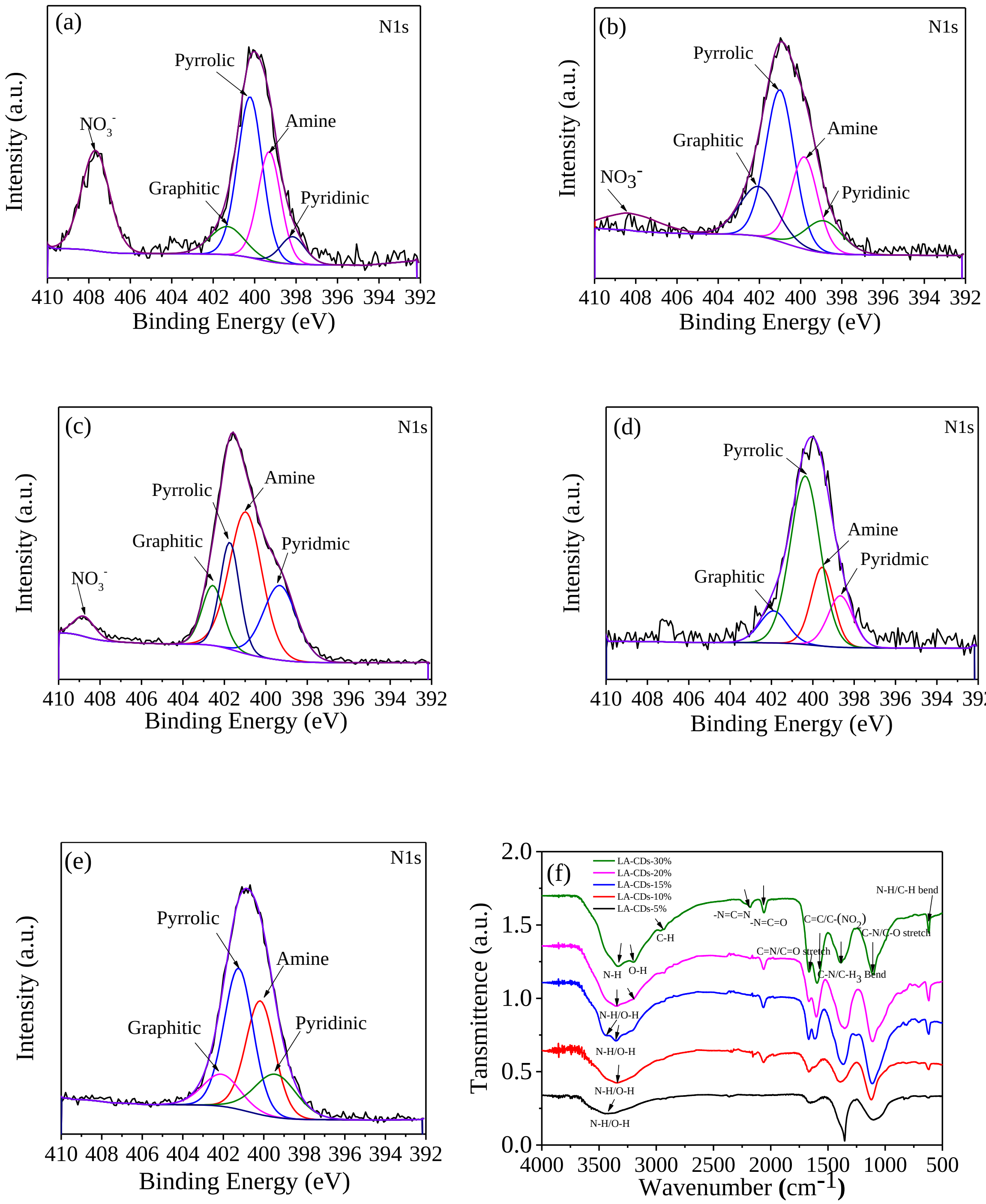

Figure S3: Deconvoluted high resolution spectra of N1s: (a) LA-CDs-5%, (b) LA-CDs-10% NH_4OH, (c) LA-CDs-15%, (d) LA-CDs-20%, and (e) LA-CDs-30%; (f) FTIR spectra of LA-CDs-x%.

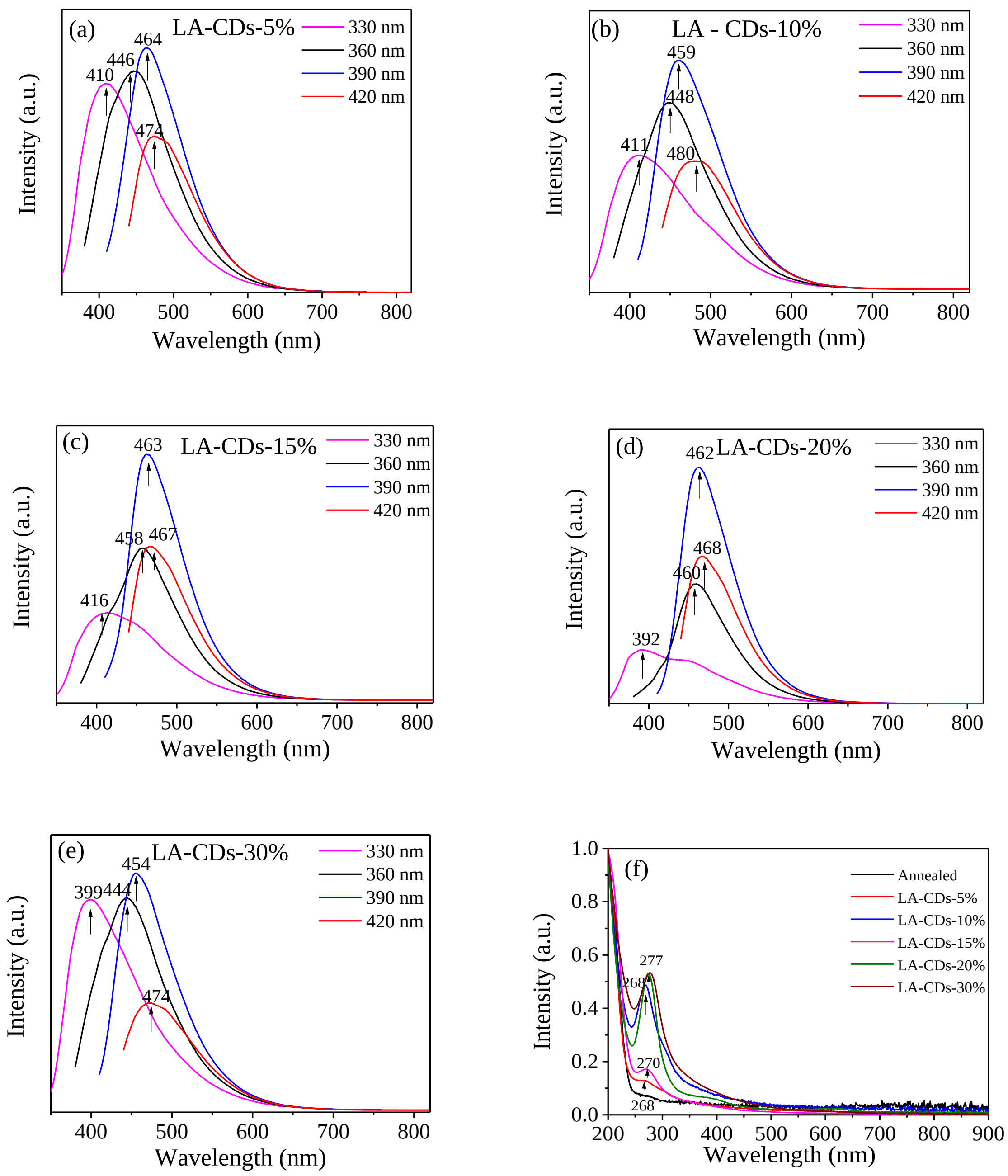

Figure S4: PL spectra of LA-CDs-x%: (a) x = 5, (b) x = 10, (c) x = 15, (d) x = 20, and (e) x = 30; (f) UV–visible absorption spectra of LA-CDs-x%.

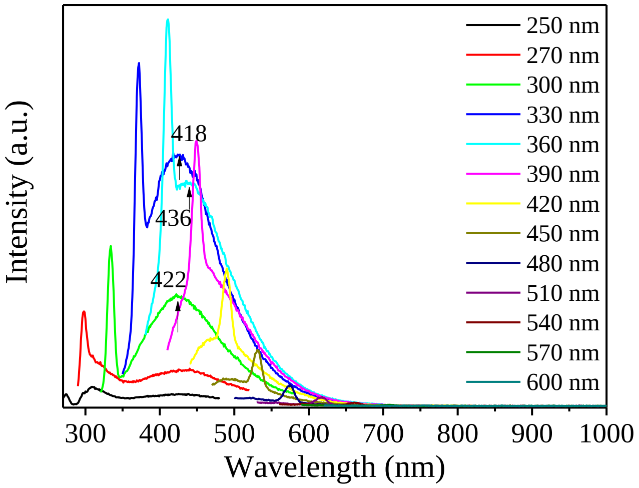

Figure S5: PL spectra of laser-ablated Teflon in deionized water.

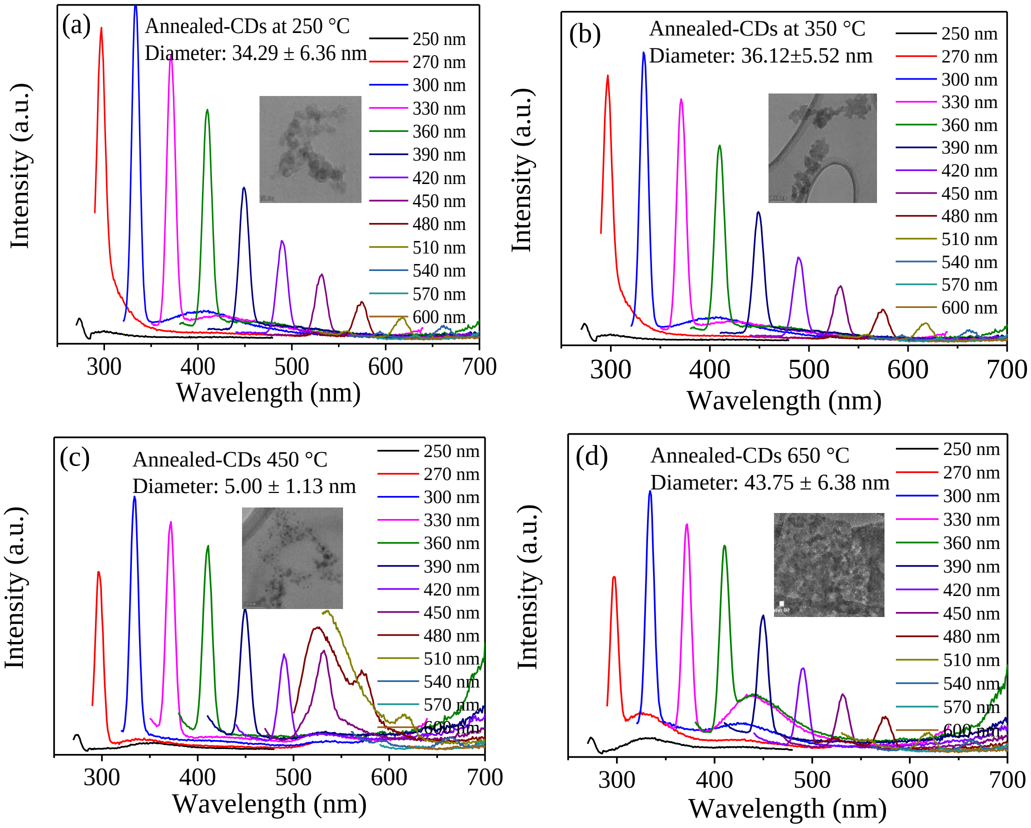

Figure S6: PL spectra of annealed soybean at different temperatures with the flow of argon gas for 2 h.

Table S1: Element fractions in the soybean-derived carbon nanoparticles calculated from the XPS spectra.

	C 1s	N 1s	O 1s
HTC-CDs	73.6	10.3	16.1
Annealed-CDs	60.0	0	40.0
LA-CDs-10%	51.1	4.4	44.5

Table S2: Element fractions in LA-CDs-x% calculated from the XPS spectra.

Element	LA-CDs-5%		LA-CDs-10%		LA-CDs-15%		LA-CDs-20%		LA-CDs-30%	
	Position (eV)	atom %	Position (eV)	atom %	Position (eV)	atom %	Position (eV)	atom %	Position (eV)	atom %
C	285.4	42.6	286.4	51.1	287.2	54.7	285.2	38.9	287.9	60.1
N	400.1	3.8	400.8	4.4	401.4	7.8	400.3	4.7	402.1	5.2
O	532.7	53.6	533.1	44.5	533.4	37.5	532.4	56.4	534.6	34.7

Table S3: Area ratios of bond structures in LA-CDs-x% calculated from the XPS spectra.

Name/Area Ratio%	Graphitic	Pyrrolic	Amine	Pyridinic
LA-CDs-5%	8.3	49.4	30.9	8.4
LA-CDs-10%	19.0	44.0	25.1	11.9
LA-CDs-15%	12.3	19.8	44.1	23.8
LA-CDs-20%	10.1	55.9	19.5	14.5
LA-CDs-30%	11.4	39.4	31.9	17.3

Absolute Quantum yield (QY) measurement with integrating sphere: The QY was measured and calculated on Fluoromax-4 using integrating sphere according to the following equation as below:

$$QY = \frac{\text{Number of emitted photos}}{\text{Number of absorbed photos}} = \frac{P_b - P_a}{L_b - L_a}$$

L_b = Total number of incident photons, L_a=Total photos not absorbed by sample.

P_a = “Dark signal” in the emission wavelength area, P_b=Total number of photons emitted in emission wavelength area.
